# The Use of Directed Evolution to Create a Stable and Immunogenic Recombinant BCG Expressing a Modified HIV-1 Gag Antigen

**DOI:** 10.1371/journal.pone.0103314

**Published:** 2014-07-25

**Authors:** Rosamund Chapman, William R. Bourn, Enid Shephard, Helen Stutz, Nicola Douglass, Thandi Mgwebi, Ann Meyers, Nyasha Chin'ombe, Anna-Lise Williamson

**Affiliations:** 1 Division of Virology, Institute of Infectious Disease and Molecular Medicine, Faculty of Health Sciences, University of Cape Town, Cape Town, South Africa; 2 Medical Research Council, Cape Town, South Africa; 3 Department of Molecular and Cell Biology, Faculty Of Science, University of Cape Town, Cape Town, South Africa; 4 National Health Laboratory Services, Cape Town, South Africa; 5 Department of Medicine Faculty of Health Sciences, University of Cape Town, Cape Town, South Africa; University of Delhi, India

## Abstract

Numerous features make *Mycobacterium bovis* BCG an attractive vaccine vector for HIV. It has a good safety profile, it elicits long-lasting cellular immune responses and in addition manufacturing costs are affordable. Despite these advantages it is often difficult to express viral antigens in BCG, which results in genetic instability and low immunogenicity. The aim of this study was to generate stable recombinant BCG (rBCG) that express high levels of HIV antigens, by modification of the HIV genes. A directed evolution process was applied to recombinant mycobacteria that expressed HIV-1 Gag fused to the green fluorescent protein (GFP). Higher growth rates and increased GFP expression were selected for. Through this process a modified Gag antigen was selected. Recombinant BCG that expressed the modified Gag (BCG[pWB106] and BCG[pWB206]) were more stable, produced higher levels of antigen and grew faster than those that expressed the unmodified Gag (BCG[pWB105]). The recombinant BCG that expressed the modified HIV-1 Gag induced 2 to 3 fold higher levels of Gag-specific CD4 T cells than those expressing the unmodified Gag (BCG[pWB105]). Mice primed with 10^7^ CFU BCG[pWB206] and then boosted with MVA-Gag developed Gag-specific CD8 T cells with a frequency of 1343±17 SFU/10^6^ splenocytes, 16 fold greater than the response induced with MVA-Gag alone. Levels of Gag-specific CD4 T cells were approximately 5 fold higher in mice primed with BCG[pWB206] and boosted with MVA-Gag than in those receiving the MVA-Gag boost alone. In addition mice vaccinated with BCG[pWB206] were protected from a surrogate vaccinia virus challenge.

## Introduction

The estimated HIV prevalence rate in South African adults is still unacceptably high at 17.9%, and is as high as 30% in some regions[1], despite continuing government efforts to extend antiretroviral therapy and other interventions. Thus, a safe and effective HIV vaccine is required to significantly reduce the HIV infection rate. Within the HIV vaccine scientific community there is renewed optimism about the prospects of success, following the first successful HIV vaccine efficacy trial in Thailand (RV144), in which a canarypox vector prime and a gp120 protein boost were utilised. Although there was only a 31% reduction in the rate of HIV infection in vaccinated people compared with those receiving placebo, it is the first indication that vaccination against HIV acquisition is possible [2].

While antibody responses are important in preventing HIV infection, there is broad agreement that a robust T cell response is also desirable; the aim of this is to control HIV infected cells if the antibodies fail to prevent infection [3]–[5]. In our laboratory we are attempting to use the live, anti-tuberculosis vaccine *Mycobacterium bovis* BCG to make a recombinant vaccine that delivers HIV-1 subtype C antigens, the subtype responsible for the devastating epidemic in sub-Saharan Africa. BCG has many features that make it a promising vaccine vector: it has been administered to billions of people worldwide with a very low incidence of complications; it is cheap to manufacture; it elicits long-lasting cellular immunity and it is unaffected by maternal antibodies. A variety of viral, bacterial, parasitic and human antigens have been successfully expressed in BCG. In experimental models rBCG has elicited protective immunity against a variety of infectious agents including viruses (measles, papillomavirus), bacteria (listeriosis, pneumococcal infection, pertussis, Lyme disease) and parasites (leishmaniasis and malaria) [6]–[13]. This work is promising, however, the weaknesses of the system are generally not addressed. A major limitation is the fact that recombinants can be unstable [14]–[19]. This manifests as mutations within the recombinant DNA, resulting in the loss of, or a decrease in, cloned antigen expression or, in the absence of selection, in the loss of the recombinant plasmid. This is a serious defect, because such mutants cannot be selected against during scaled-up production, precluding the vaccine from commercial development.

For antigen expression to fail, a mutation in the recombinant gene must occur. This probably occurs in a small fraction of the population in all cultures and is unimportant, provided the mutants do not have a selective advantage, typically a higher growth rate. This superior growth is achieved by elimination or lower expression of the cloned gene, which otherwise imposes some form of metabolic or toxic load on the parent strain [20]. Researchers have attempted to solve the instability problem by utilising promoters that, during laboratory or commercial culture, express weakly or not at all, but which express strongly after vaccination [21]. Alternatively, researchers have attempted to stabilise recombinant DNA by using single-copy, integrative vectors, as opposed to multi-copy plasmid vectors [22]. While this appears to work to some degree, it is probable that this is partially due to a decrease in expression of the foreign gene and consequent decrease in the metabolic or toxic load.

Efforts to improve the level of antigen expression in BCG have focused on methods of increasing the amount of *de novo* antigen synthesis. These include the use of multi-copy vectors, strong expression signals and genes with corrected codon bias [17]. However, as this increases the burden on the bacteria, such approaches must inevitably result in increased instability. In the work here, we demonstrate that poor expression and instability are causally related. We argue that instability and poor expression occurs primarily in cases where the recombinant antigen is of eukaryote origin, and is due to miss-folding of the protein. When such miss-folding occurs, the host bacterium presumably responds by up-regulating the components of the protein quality control apparatus (the heat shock response), mainly chaperones and proteases [23]. As there is no reason to expect that the chaperones will be able to fold the antigen in a manner acceptable to the bacterium, the proteases would be expected to destroy it. This would explain both the instability (caused by stress and slow growth) and low expression (caused by antigen destruction). This insight has allowed us to devise a method whereby antigens can be expressed at 14 fold higher levels while at the same time stability is improved.

In this study we aimed to improve the stability and immunogenicity of HIV-1 Gag- expressing rBCG by applying a directed evolution process. This involved modifying the antigen by mutagenesis and then selecting for HIV-1 Gag-expressing recombinant mycobacteria that show both higher growth rates and increased antigen expression. Such a process should yield rBCG that express modified versions of Gag that are less deleterious, possibly because of a more acceptable manner of protein folding. We have identified rBCG that are more stable and have higher Gag antigen expression levels than the parent rBCG. Upon vaccination one of these recombinants induced improved immune responses in a mouse model.

## Methods

### Bacterial strains, culture, transformation, preparation of vaccines

Bacterial strains *Escherichia coli* LKIII [24] *E. coli* Top10, (Invitrogen), *Mycobacterium smegmatis* mc^2^155 [25] and *Mycobacterium bovis* BCG (Pasteur) were used. *E.coli* was cultured at 37°C in Luria-Bertani broth or on agar [26] with kanamycin 15 µg/ml as appropriate. *M.smegmatis* and *M. bovis* BCG were cultured at 37°C in Middelbrooks 7H9 broth (Difco) supplemented with oleic acid-albumin-dextrose-catalase (OADC) enrichment, 0.2% glycerol and 0.05% Tween 80 or 0.025% tyloxopol in roller bottles or on Middelbrooks 7H10 (Difco)/OADC agar with kanamycin 10 µg/ml when appropriate. Liquid culture growth and cell density of bacterial cultures were monitored by recording the absorbance at 600 nm. Electrotransformation of *M. bovis* BCG, *M. smegmatis* and *E. coli* was as previously described [27].

Vaccine stocks of all BCG recombinants were prepared by culture in Middelbrooks 7H9 broth supplemented with OADC, 0.2% glycerol and 0.025% tyloxopol [16]. Plasmid DNA was isolated from all recombinant BCG vaccine stocks and restriction enzyme mapped and the *gag* gene was sequenced to confirm the integrity of the plasmids.

Recombinant MVA expressing Gag (MVA-Gag) was grown on the chorioallantoic membranes of 10–12 day-old chick embryos and harvested after 72 hours [16]. This work was carried out according to the guidelines and approval of the UCT Animal Research Ethics Committee.

### Plasmids

**Figure 1:**
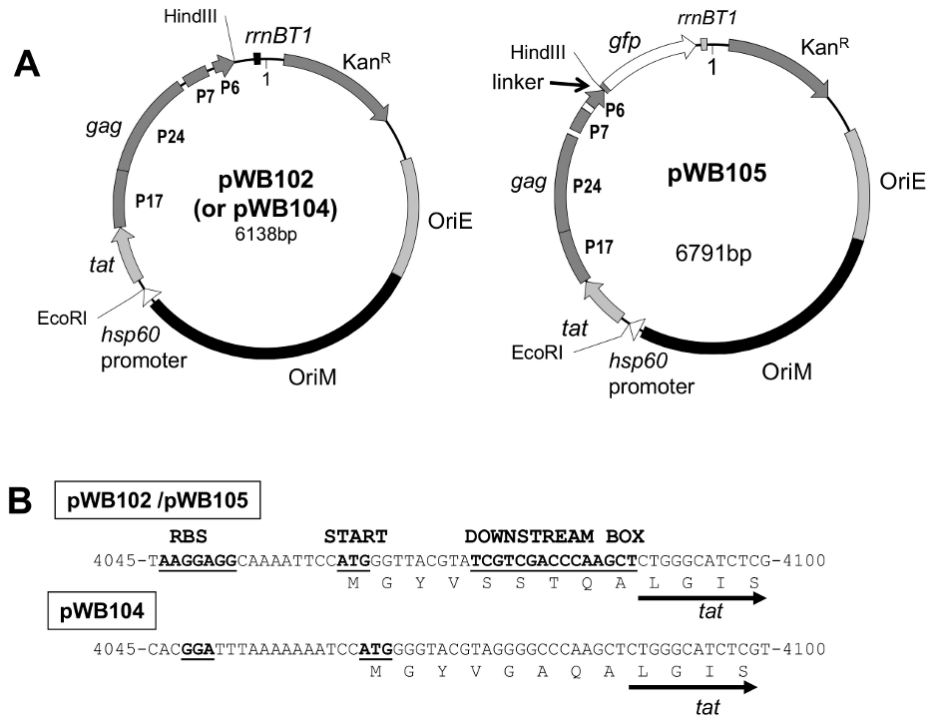
Schematic map and details of the expression cassette and translation control signals of *E. coli-*mycobacterial shuttle vectors. A. Schematic map of plasmids pWB102 and pWB105: *hsp60*, the mycobacterial promoter; RBS, ribosome binding site; *tat* and *gag*, the translationally fused *tat* and *gag* genes; *rrnBt1 E. coli* transcription terminator; Linker sequence of A,L and S codons; *gfp*, gene encoding the green fluorescent protein. B. Details of translation control signals. The ribosome binding site, start codon and downsteam box are bold and underlined. Arrow indicates the beginning of the *tat* coding sequence. Nucleotide positions are as recorded in Genbank.

All *E. coli*-mycobacterial shuttle vectors are kanamycin resistant, with designs based on the pMV261-type mycobacterial expression systems [28] and were created for this work by standard gene cloning methods [26]. Plasmid pWB100 is a control plasmid that carries no expression cassette (accession number DQ191755). The high-copy-number mutant mycobacterial plasmid pHIGH100 has been described ([29], EF216316). Plasmids pWB102 (EF216320), pWB104 (EF216321) and pWB105 (EF216322), are described in Figure 1. Plasmids pWB106 (EF216324), pWB206 (EF216325), pWB107 (EF216326) and pHS207 (EF216327) were isolated after directed evolution (below). Plasmids pWB102 and pWB104 are expression vectors that carry modified HIV-1 subtype C genes *tat* and *gag* as a translational fusion. Both genes have been codon optimised for BCG and the *tat* gene has been rearranged to disrupt its activity [30]. Transcription of the gene fusion is driven by the mycobacterial *hsp60* promoter. In plasmid pWB102, translation of the recombinant protein is driven by a powerful consensus Shine-Delgarno sequence and downstream box, with the correct spacing for maximum expression, whereas plasmid pWB104 contains much weaker translational signals [31], [32]. In addition to this, the vectors carry a transcription terminator downstream of the HIV genes, a kanamycin resistance gene, an *E. coli* plasmid origin of replication and a mycobacterial plasmid origin of replication.

### Isolation and enzymatic treatment of DNA

Unless otherwise stated, standard enzymatic and other treatments of DNA were as recommended by Sambrook *et al*. [26] or the manufacturer. Isolation of plasmid DNA from mycobacteria was as described by Parish and Stoker [27].

### Protein isolation and p24 capture ELISA

Total protein was isolated using the SDS boiling method [33]. To determine the protein concentration a *DC* protein assay kit (Biorad) was used with bovine serum albumin (Roche, molecular biology grade) as the standard. Capture ELISA was conducted using the automated Elecsys 2010 (Roche) and an Elecsys HIV Ag (p24) kit with purified recombinant His-tagged-P24 [34] as the standard.

### Directed evolution

In order to create a pool of mutants that carry small deletions and insertions, 4 µg samples of the plasmid pWB105 were digested with dilutions of DNase 1 (Roche, molecular biology grade) 0.0125–0.0016 U/µg in DNase 1 buffer (10 mM MnCl_2_, 50 mM Tris-Cl, pH 7.6,) at 37°C in 100 µl. In the presence of manganese, DNAseI cuts the target at random positions and leaves a mixture of both 3′- and 5′- overhanging ends, of different lengths. Reactions were stopped by the addition of 20 µl of 0.25 M EDTA and phenol chloroform extraction. DNA was precipitated with isopropanol and resuspended in TE buffer. Aliquots were visualised on an agarose gel. Linearised plasmid containing samples, were pooled and blunt ends generated with T4 DNA polymerase, this results in either a small deletion or duplication, depending upon the nature of the overhang. The sample was separated on an agarose gel. The full-length and near-full-length linearised plasmids were isolated by electroelution, and purified DNA was resuspended in 180 µl of ligation mix for re-circularised by ligation with T4 DNA ligase (Roche). Batches of *E. coli* LKIII were CaCl_2_-transformed with 5 µ µl aliqots of the ligation mix. Following transformation samples were immediately pooled, and aliquots removed for plate counts. The remainder of the mixture was cultured in broth under kanamycin selection and plasmid DNA was isolated.

Starting with that pool of deletion/insertion mutants, and using primers 5′-CTAAGAATAACGTTGGCACTCG-3′ and 5′-TTTGTGCCCATTAACATCACC-3′ the region between nucleotides 4001 and 6038 (numbering according to Figure 1) was amplified by error-prone PCR, in order to create point mutations. Taq DNA polymerase (Promega) was used with the template at 20 fg/µl, and 30 cycles of priming at 54°C for 30 sec and extension at 72°C for 90 sec, all other conditions were as recommended. The high cycle number and the fact that the nucleotide mix was equimolar and did not reflect the G+C richness of the target promote the likelihood of errors occurring. Equal amounts of both the mutant pool and original pWB105 plasmid DNA were used as templates. After purification, the PCR product was digested with *Eco*RI and *Hin*dIII, separated on an agarose gel and the 1.9 kb internal *Eco*RI-*Hin*dIII fragment (nucleotides 4022–5929) was isolated. The *Eco*RI-*Hin*dIII fragments were then ligated with *Eco*RI-*Hin*dIII digested pWB105, which had been dephosphorylated, back into their original position to create a library of mutants. The ligation mix was purified and used to transform electrocompetent *E. coli* LKIII. Following transformation, aliquots were removed for plate counts. The remainder of the transfomants were broth-cultured. Plasmid DNA was isolated from this pool of mutants and used to transform *M. smegmatis*. Transformants were selected on solid medium. Colonies were viewed under UV light and bright fluorescing clones were streaked onto fresh solid medium. After incubation, colony sizes were compared to those of similarly treated *M. smegmatis*[pWB105]. Clones were also patched onto solid medium and, after culturing, cells from each patch were removed from the plate, total protein was isolated and the level of Gag p24 assessed by capture ELISA. This was compared to similarly treated *M. smegmatis*[pWB105]. Clones that displayed 2-fold or higher levels of Gag p24 and equivalent or better growth, as estimated from colony size, than the parental strain were selected.

Plasmid DNA was isolated from each of the promising clones, and the plasmids were used to transform *E. coli*. Plasmid DNA was isolated and restriction endonuclease mapped. *E. coli* that carried plasmids with gross deletions were discarded. The remaining recombinants were pooled, cultured and plasmid DNA was isolated on a large scale.

To shuffle and introduce further mutations 80 µg of the plasmid pool was first linearised by digestion with *Xba*I then digested (10 µg DNA) with DNase 1 (0.007–0.0002 U/µg in 20 µl, 37°C, 30 min). Reactions were stopped as previously described. DNA from each sample was separated and visualised on an agarose gel. Fragments of 100–800 bp were isolated, pooled and subjected to error prone assembly PCR as follows: A standard PCR mixture was assembled in 20 µl, except no oligonucleotide primers were included. The thermo-cycle consisted of a 30 sec, 95°C denaturation step, and a 2 min, 72°C extension step which were repeated 45 times, following which 5 µl 0.25 M EDTA was added to the reaction. The DNA was then purified and digested with *Cla*I and *Xba*I, separated on an agarose gel and the 2–4 kb size fraction purified. Error prone PCR was conducted as in the first round of mutagenesis. The PCR product was digested and the *Eco*RI-*Hin*dIII fragments were inserted back into their original position in pWB105 to create an *E. coli* library of mutants as before. DNA was isolated from this pool and used to transform *M. smegmatis*; colonies were again viewed under UV light and selected by p24 expression level and colony size assessment as before.

### rBCG antigen expression and growth rate

To assess stability of recombinant *M. smegmatis* and Gag p24 antigen expression, duplicate cultures were passaged daily for 10 days (approximately 40 generations) in liquid media with and without antibiotic selection. Starter cultures were derived from glycerol stocks of a single colony of *M. smegmatis* transformants grown to an OD_600_ of approximately 1 in 10 ml liquid media. Cultures were sub-cultured at an OD_600_ of between 0.7 and 1 approximately every 24 hours. The approximate generation of each culture was calculated according to the equation: Generation  =  [ln(final OD_600_)-ln(starting OD_600_)]/ln2 [35]. Prior to each passage total protein was isolated using the SDS boiling method [33]and Gag p24 concentrations were determined. An aliquot of culture, grown in the absence of kanamycin, from days 1, 3, 6 and 10 was also diluted (10^−5^–10^−6^) and plated on solid media with and without kanamycin, to determine CFUs. Gag p24 and GFP in extracts were detected by western blot using HIV-1 Gag p24-specific antiserum (ARP432, NIBSC Centralised Facility for AIDS reagents, MRC, UK) and anti-GFP (Roche) respectively. Proteins were detected with anti-rabbit or anti-mouse alkaline phosphatase-conjugated secondary antibodies (Sigma). Purified, recombinant p24 [34] and GFP (BD Biosciences) were used as positive controls.

In order to visualise the distribution of the GFP-antigen fusions within the cells, mycobacteria that had been grown to mid-exponential phase in broth culture were viewed using an inverted epifluorescence microscope (Carl Zeiss Axiovert 200 M). The excitation and detection of GFP were performed using a FITC filter set.

### Mouse vaccinations and splenocyte preparation

All experiments were performed with female BALB/c mice (8–10 weeks old) in groups of 10 according to the guidelines and approval of the UCT Animal Research Ethics Committee. Immune responses to the rBCG constructs BCG[pHS207], BCG[pWB105], BCG[pWB106] and BCG[pWB206], at a dose of 10^7^ CFU in 50 ul, instilled in 25 µl aliquots per nostril, over a period of 10 seconds, were evaluated at 8 weeks. Intraperitoneal vaccinations with BCG[pHS207] and BCG[pWB206] at doses of 10^7^, 10^5^ or 10^3^ cfu in 200 ul were used to prime mice before a boost with MVA-GagC which encodes a matching Gag antigen. MVA-Gag (10^7^ pfu) was given to half the mice in each group as an intramuscular injection in a final volume of 100 µl with 50 µl injected into each hind leg muscle on day 56 after the rBCG prime. Immune responses were evaluated on day 68. At the end of the vaccination periods spleens from each group were pooled and a single cell suspension of splenocytes was prepared. After red blood cell lysis (0.15 M NH_4_Cl, 10 mM KHCO_3_, 0.1 mM Na_2_EDTA, 1 min at room temperature) splenocytes were suspended in R10 medium (RPMI-1640 with 10% heat inactivated FCS, 15 mM β-mercaptoethanol, 100 U penicillin and 100 µg streptomycin per ml).

### IFN-γ ELISPOT assays and quantification of cytokines

The Mouse IFN-γ ELISPOT set (BD Pharmingen) was used according to manufacturer's instructions [36], [37]. Splenocytes were plated in triplicate at 500 000 cells/well in a final volume of 200 µl R10 medium either alone to determine background responses, or in medium containing 4 µg/ml of specific peptides (>95% pure, Bachem, Switzerland). The peptides comprised either the GFP H-2K^d^-restricted CD8 T Cell peptide HYLSTQSAL or the H-2K^d^-restricted Gag CD8 peptide AMQMLKDTI or a pool of the Gag MHC class II-restricted CD4 peptides NPPIPVGRIYKRWIILGLNK and FRDYVDRFFKTLRAEQATQE. Reactions were stopped after incubation for 22 hours at 37°C in 5% CO_2_ and spots were detected using Nova Red substrate (Vector Labs) then scanned and counted using a CTL Analyzer (Cellular Technology, OH, USA) with Immunospot Version 3.2 software. The mean number of spots from triplicate wells ± standard deviation (SD) was calculated and background spots (not more than 20±10 sfu/10^6^ splenocytes) were subtracted. Data are presented as sfu/10^6^ splenocytes ± SD.

Pooled splenocytes at a concentration of 7.5×10^6^ per ml R10 culture medium were also cultured in triplicate either alone to determine background cytokine release or with the peptides as for the IFN-γ ELISPOT assay. Cytokines secreted into the supernatant were assayed using a Th1/Th2 Cytokine Bead Array assay (BD Pharmingen). IFN-γ, TNF-α, IL-4 and IL-10 levels are presented as pg cytokine per 10^6^ splenocytes.

### Generation of GagCD8 peptide-specific effector cells and ^51^Chromium-release assay

A standard ^51^Chromium-release assay was performed with GagCD8 peptide-specific effector cells generated by culturing pooled splenocytes (10^7^/ml R10) with the Gag CD8 peptide, AMQMLKDTI, at a concentration of 4 µg/ml for 6 days [38]. Harvested effector cells were incubated with ^51^Cr labelled P815 target cells at ratios of 200∶1–6∶1 in the absence and presence of the GagCD8 peptide. Supernatants were assayed for ^51^Cr release after a 4 h culture. The percentage of specific ^51^Cr release was calculated as 100× (experimental cpm-spontaneous cpm)/(total cpm-spontaneous cpm).

### Vaccinia virus challenge

The New York City Board of Health strain of vaccinia virus (NYCBH) and a recombinant vaccinia virus vT369 (VVGag C), which expresses HIV-1 DU422 Gag (Clade C), were obtained from the NIH AIDS Research & Reference Reagent Program, Division of AIDS, NIAD, NIH. These viruses were grown on 10–11 day old chick chorioallantoic membranes as described previously [39] and resuspended in PBS. Titration was performed in CV1 cells obtained from Highveld Biological, SA. Groups of BALB/c mice (5 per group) were immunized with a 200 µl intraperitoneal inoculation of either 2×10^6^ CFU BCG[pWB206], the vector control, BCG[pWB100] or the buffer used to resuspend the vaccines. The mice were given either a single inoculation or a triple inoculation at 4 week intervals. Two weeks after the final inoculation half the mice in each group were challenged with 1×10^6^ pfu VVGag C and the other half challenged with 1×10^6^ pfu control NYCBH, given as a 200 µl intraperitoneal injection. Five days after challenge all mice were killed by cervical dislocation and ovaries collected bilaterally. Crude extracts of virus were prepared from the ovaries by chopping the ovaries, placing them in <5 ml McIlvain's solution (4 mM citrate phosphate buffer, pH 7.4), performing 30 strokes in a tenbrook grinder, freeze/thawing three times and then subjecting the homogenate to centrifugation at low speed for 5 minutes to remove cell debris. The volumes were adjusted to 5 ml (1 ml per mouse) prior to centrifugation. Titrations were performed in CV-1 cells in triplicate. Thirty six hours post infection cells were stained using carbol fuschin and plaques were counted. Titres were expressed as pfu/ml which was equivalent to pfu/mouse.

## Results

### The effect of HIV -1 Gag antigen expression on the stability and growth rate of recombinant mycobacteria

**Figure 2:**
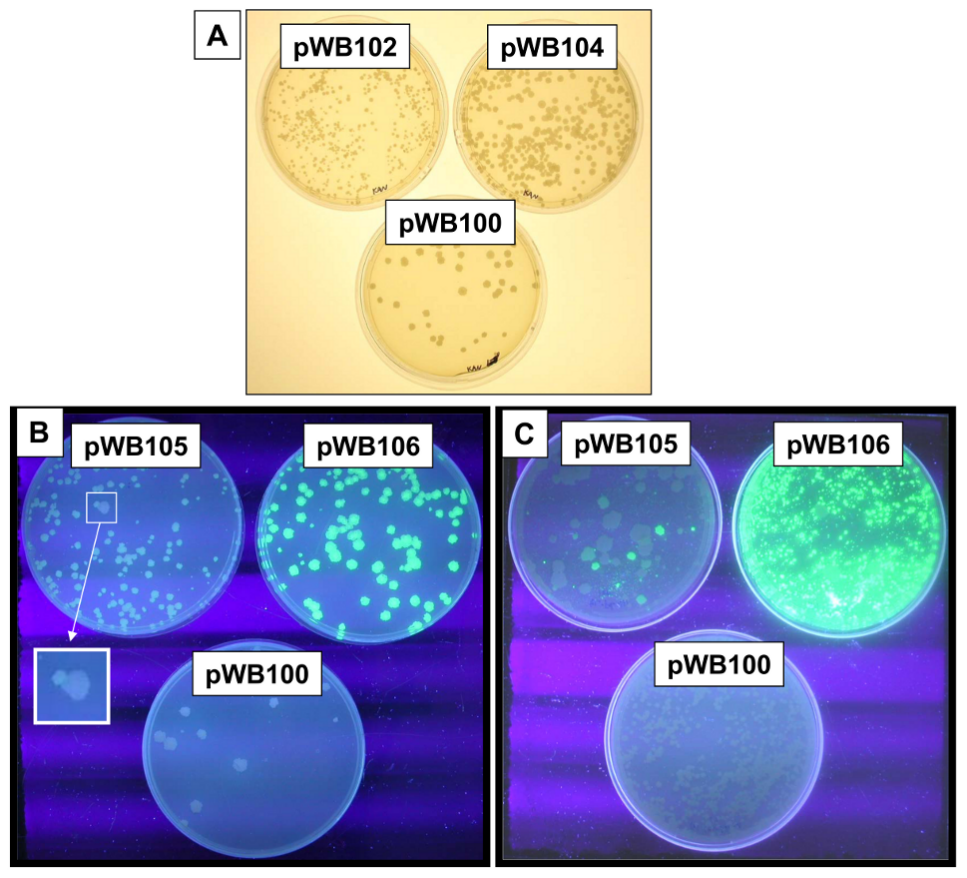
Colony size and expression of GFP by recombinant mycobacteria grown on solid medium. **A.** Colonies of *M. smegmatis* transformed with pWB102, pWB104 and pWB100 viewed under normal light. **B.** Colonies of *M. smegmatis* transformed with pWB100, pWB105 or pWB106 viewed over UV light. The insert shows a magnified view of a mutant, non-expressing clone adjacent to a normal clone. C. Colonies of *M. bovis* BCG transformed with either pWB100, pWB105 or pWB106 viewed over UV light.

The relationship between mycobacterial growth and level of HIV Gag antigen expression was investigated using the fast-growing model organism *M. smegmatis*. Relative growth rates of *M. smegmatis*[pWB102], *M. smegmatis*[pWB104] and [pWB100] were compared four days after transformation. Translation of the Tat-Gag fusion protein in plasmid pWB102 (Figure 1, EF216320) is driven by a powerful consensus Shine-Delgarno sequence and downstream box, with the correct spacing for maximum expression. The second plasmid, pWB104 (Figure 1, EF216321) is identical to the first, except that the powerful Shine-Delgarno and downstream box sequences have been replaced by much weaker translation signals. This should result in much lower levels of antigen synthesis. The third plasmid, pWB100, is identical to the other two, except that the *hsp60* promoter and HIV genes have been removed. All colonies of *M. smegmatis*[pWB102] were visually much smaller than *M. smegmatis*[pWB100] or [pWB104] (Figure 2A). To relate protein expression levels and colony size, two single colonies of each recombinant were picked and cultured to mid-log phase, total protein was extracted and Gag p24 content measured. Gag p24 protein levels ranged between 0.1% and 0.05% of the total mycobacterial protein in the case of *M. smegmatis*[pWB102] and was 30 fold less in the case of *M. smegmatis*[pWB104]. This growth inhibition on solid media also occurred in liquid media. *M. smegmatis*[pWB102] had a doubling time of approximately 5.4 hours in liquid culture whereas *M. smegmatis*[pWB104] had a doubling time of approximately 3.8 hours. In the absence of antibiotic selection in liquid culture Gag p24 levels in *M. smegmatis*[pWB102] extracts fell to background levels within seven generations, while Gag p24 levels for *M. smegmatis*[pWB104] dropped to half after fifteen generations and remained at this level for over 60 generations (data not shown). These results suggest that mycobacteria expressing high levels of foreign antigen experience growth retardation in both solid and liquid culture and are unstable.

This growth inhibition maybe a consequence of induction of the heat shock responses, whichoccurs as a consequence of the recombinant proteins being unable to fold correctly and/or aggregating in the bacteria and leading to the destruction of the expressed protein [40]. To demonstrate that the stress/heat shock response is induced in those mycobacteria expressing a certain level of foreign antigen, *M. smegmatis* was electroporated with plasmids [pWB102], [pWB104] and [pWB100], held at 37°C for 2 hours to allow antigen expression and then plated on solid media, grown at either 37C or 43°C and scored for colony growth after 3 days. All transformants grew at 37°C, although *M. smegmatis*[pWB102] colonies were the smallest of the three as shown before in Figure 2A. The control *M. smegmatis*[pWB100], which expresses no antigen, never survived growth at the elevated temperature of 43°C. In contrast, *M. smegmatis*[pWB102] always survived at this high temperature and only a few *M. smegmatis*[pWB104] colonies grew. Thus the *M. smegmatis*[pWB102] transformants that produced the most foreign antigen, out of the three tested, survived a heat challenge, implying that the heat shock defences had been pre-induced.

### Generation of a stable mycobacterial recombinant with improved antigen expression

The data presented above supports the contention that poor bacterial growth and antigen expression are both due to the inability of the recombinant antigen to fold correctly, which in turn induces stress responses. It can then be argued that, by improving the acceptability of the recombinant antigen to the bacterial protein quality control apparatus, higher levels of antigen expression can occur. To test this we used a method, based on the “folding reporter” system of Waldo *et al*.[41]. This system involved fusing randomly mutated antigen to the N-terminus of the green fluorescent protein (GFP). GFP fluorescence levels are dependent on both expression level and fusion-protein folding. Bright clones expressing high levels of GFP were selected. The growth rates of the recombinant bacteria were also assessed, in terms of colony size, which was assumed to be a surrogate measure of overall fitness. Fitter, high- expressing mutants were thus identified.

For the target we used *M. smegmatis*[pWB102], which showed growth retardation and low Gag-p24 accumulation. Plasmid pWB102 was modified such that a 12 amino acid linker and GFP encoding sequence was fused to the *tat-gag* recombinant gene to create pWB105 (Figure 1, EF216322). *M. smegmatis*[pWB105] fluoresced weakly under UV light and displayed growth inhibition (Figure 2B). Instability was again apparent, in that occasionally a larger colony that did not fluoresce was seen, (Figure 2B, expanded insert). *M. smegmatis*[pWB105] was subjected to directed evolution as described in the Methods. In the first round 16 clones were isolated from a library of 50000 mutants, these were pooled and used as the target for a second round of mutagenesis and creation of a library of 400000 mutants. Ultimately four transformants that expressed extremely high levels of Gag p24 and showed no growth inhibition in comparison to *M. smegmatis*[pWB100] (which expresses no HIV antigens) were isolated. The plasmids carried by these clones were designated pWB106, pF56, pF122 and pF242. Expression levels of GFP in *M. smegmatis* and rBCG transformed with plasmid pWB106 (selected clone), pWB100 (empty vector) and pWB105 (original clone) were compared by viewing the colonies under UV light (Figure 2B & C). Transformants containing the selected clone (pWB106) showed higher levels of GFP expression and approximately 13 fold higher levels of Gag p24 expression(738 pg Gag p24/µg cell lysate). They were fitter (as judged by colony size) than the parent clone, *M. smegmatis*[pWB105] (53 pg Gag p24/µg cell lysate).

**Figure 3:**
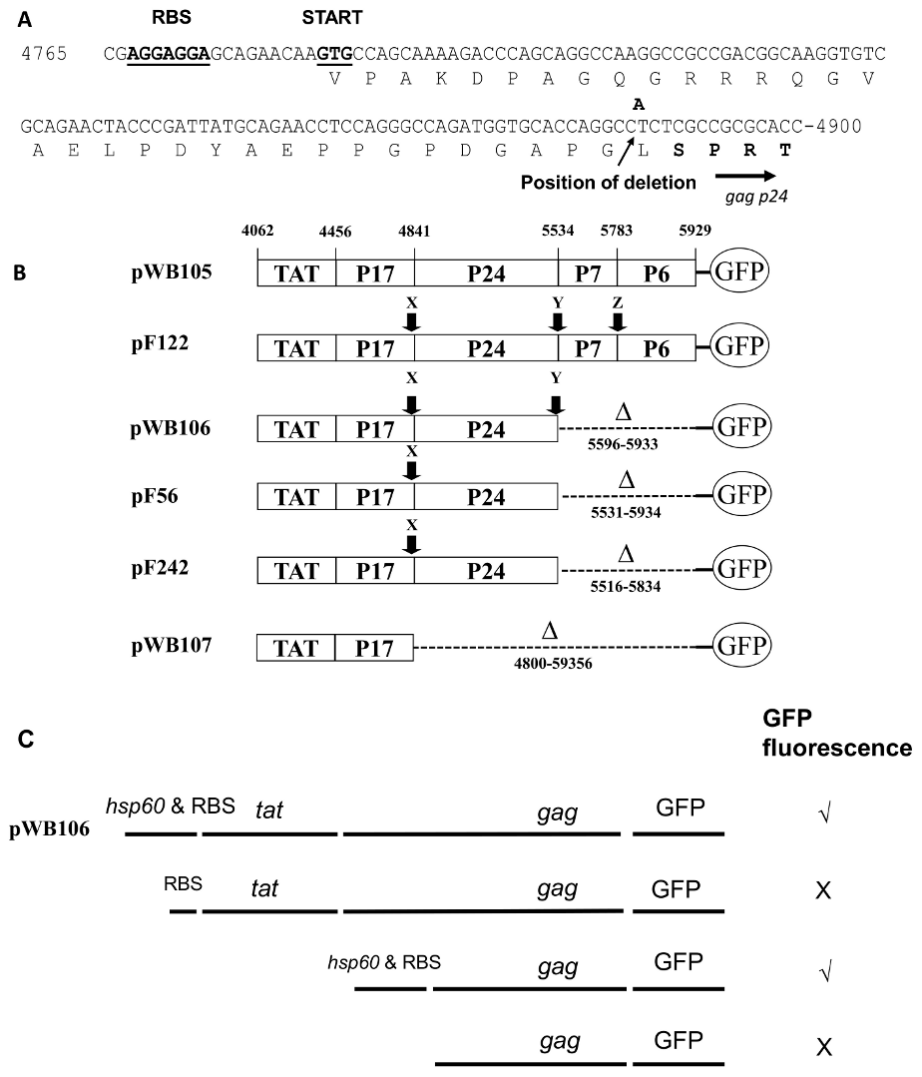
Maps and sequence of plasmids that express high levels of GFP, generated through mutation and selection. Position numbers are as for the parental plasmid pWB105.**A.** Potential ribosome binding site and start site utilized for P24-GFP expression in pWB106 transformants. **B.** Mutations found in transformants expressing high levels of p24. Mutation type symbols: X, 1 base pair deletion of an A nucleotide at position 4884 that allows expression of p24 from an internal start codon;Y, 1 bp insertion of an A nucleotide between positions 5539–5540; Z, 1 bp deletion of a C nucleotide at position 5706. Deletions (Δ) are shown with positions given to include the remaining portion. **C.** Map showing plasmids generated by deleting regions of plasmid pWB106 to determine whether p24-GFP is transcribed from the *hsp60* promoter. GFP fluorescence indicates whether *M. smegmatis* transformed with these plasmids showed GFP fluorescence.

The high GFP expressing constructs pWB106, pF56, pF122 and pF242 were sequenced and similar yet different mutations were detected. In every case there had been a 1 base pair deletion that dissociated translation of *tat*-*gag* p17 and *gag* p24 such that p24 was expressed from a fortuitous internal ribosome-binding site and GTG start codon (Figure 3A). For pWB106, pF56 and pF242, p7 and p6 were completely deleted, in the other (pF122), the p7 coding sequence was converted into out-of-frame nonsense, while p6 was maintained in-frame (Figure 3B). An extremely bright clone, which carried the plasmid designated pWB107, was also isolated. In this case, the entire Gag p24-p7-p6 region had been deleted. This resulted in the GFP being expressed from the same internal start codon as for the other constructs, but without any associated Gag p24 antigen. In every case where a deletion occurred, it was such that the GFP was brought into the correct frame to allow GFP expression. To confirm that the increased Gag p24-GFP expression was due to the internal ribosome binding site-GTG start codon and not due to a new promoter generated during directed evolution, various deletions of pWB106 were created, transformed into *M. smegmatis* and GFP expression determined (Figure 3C). The constructs lacking the *hsp60* promoter showed no GFP expression in *M. smegmatis*, indicating that antigen expression is dependent on the *hsp60* promoter.

**Figure 4:**
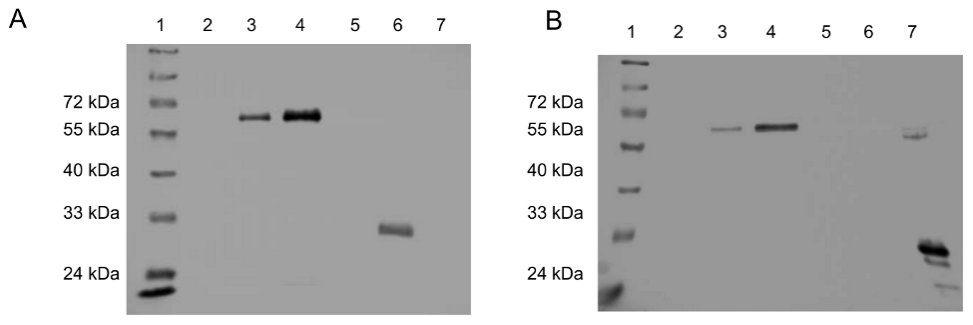
Western blots of antigen expressed by recombinant BCG. **A.** Detection with anti-GFP antibodies. **B.** Detection with anti-p24 antibodies: Lanes; 1, molecular size marker; 2, pWB105; 3, pWB106; 4, pWB206; 5, pHS200; 6, pHS207; 7, purified recombinant p24 protein.

The low-copy-number mycobacterial plasmid replicon (consisting of the origin of replication and replication proteins) was replaced with a high-copy-number mutant version of the same replicon. The mutant replicon displays a 7-fold increase in copy number and this conversion can result in stronger expression of cloned genes [29]. The plasmids pWB100 (empty vector), pWB105 (parental antigen expression), pWB106 (strongest Gag p24 expression) and pWB107 (GFP only expression) were thus converted, to create pHS200, pWB205, pWB206 and pHS207 respectively. The plasmids were used to transform *M. smegmatis* and BCG. Colony sizes and Gag p24 expression levels were determined. Expression of the cloned antigen was shown by capture ELISA to have increased approximately 3 fold (*M.smegmatis*[pWB105]  = 53, [pWB106]  = 738, [pWB205]  = 332, [pWB206]  = 2268 pg Gag p24/µg cell lysate). This increase in cloned antigen expression was confirmed by Western blotting of protein extracts (Figure 4). As expected, antigen could not be detected in the case of BCG[pWB105], but was present and showed little degradation in the case of BCG[pWB106] and BCG[pWB206], which displayed the highest level of expression.

On solid media, *M. smegmatis*[pWB205] displayed severe growth inhibition, while *M. smegmatis*[pWB206] was fitter (colonies were larger) than *M. smegmatis*[pWB205], but less so than *M. smegmatis*[pWB106]. *M. smegmatis*[pHS200] displayed no growth inhibition when compared to pWB100 (data not shown). In BCG, plasmid pWB206 caused strong expression of Gag p24 and GFP while the plasmid pWB205 was lethal. Only rBCG containing mutant plasmids of pWB205 were obtained, and then only if very large numbers of transformants were plated.

**Figure 5:**
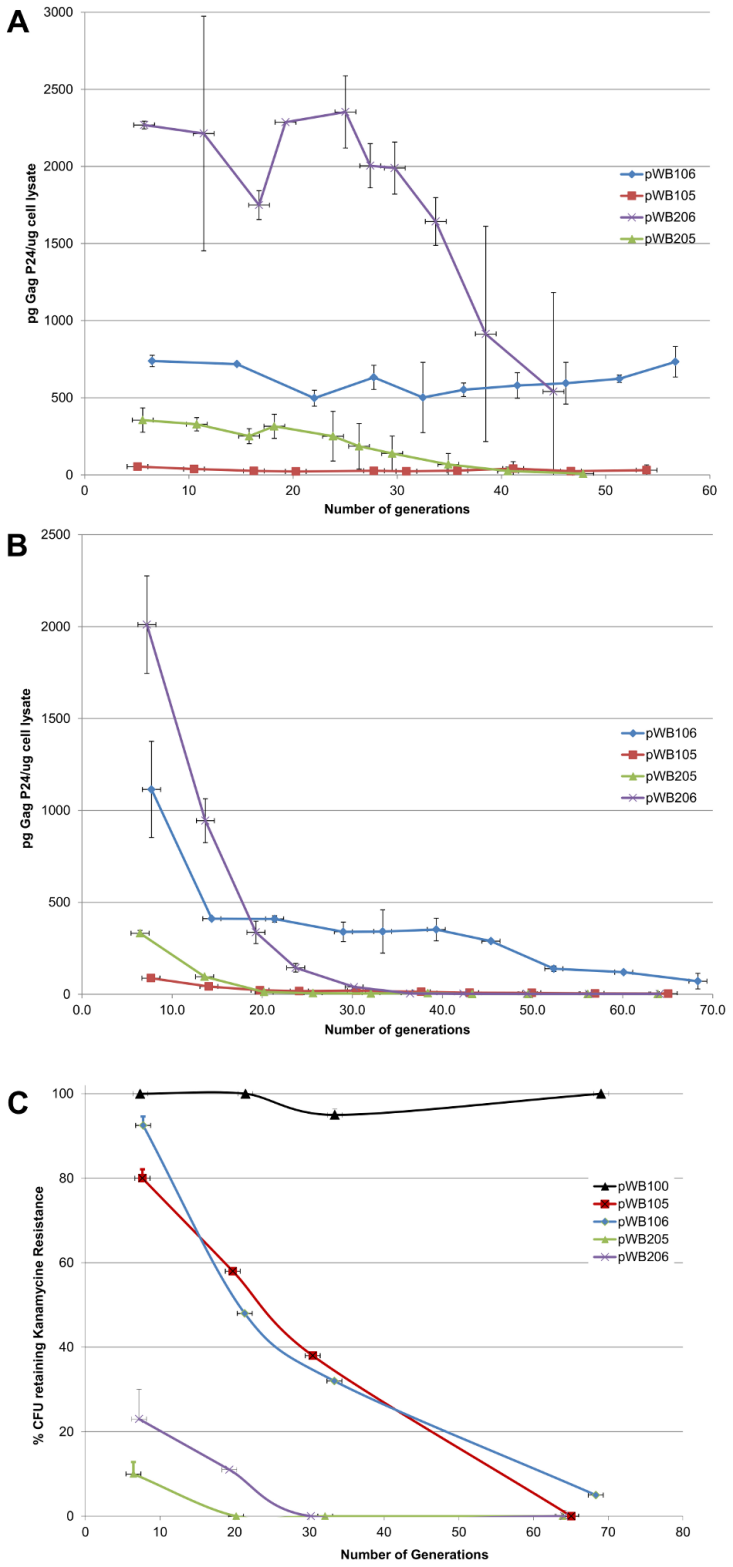
Stability of recombinant *M. smegmatis*. Duplicate cultures of recombinant *M. smegmatis* were passaged daily for 40 generations in liquid media with **A.** or without **B.** antibiotic selection. Expression of p24 in cell-free lysates was measured by capture ELISA. **C.** The % CFU retaining antibiotic resistance when passaged without antibiotic selection was determined by plating suitable dilutions of the cultures on solid media with and without kanamycin.

The stability, or ability of recombinant *M. smegmatis* to maintain Gag p24 expression over multiple generations of liquid culture, was therefore assessed. Duplicate cultures were passaged for over 40 generations, total protein was extracted before each passage and Gag p24 content determined. In the presence of antibiotic selection, the level of Gag p24 in the extracts of the parent *M. smegmatis*[pWB105] and the high copy number version *M. smegmatis*[pWB205] was low prior to passage and completely lost by 40 generations (Figure 5A). In contrast extracts of the selected clone *M. smegmatis*[pWB106] maintained initial high levels of Gag p24 for over 40 generations. Large error bars can be seen for *M. smegmatis*[pWB206] cultures after approximately 35 generations as Gag p24 levels in one of the duplicate cultures had dropped to approximately 4% of the original level by 40 generations. Whereas Gag p24 levels of the second culture had only dropped to 44% of the original level after 40 generations (Figure 5A). As each 10 generations translates into an approximate 1000-fold increase in bacterial numbers, passaging for 40 generations can result in a 10^12^-fold increase of the original starter culture. In the absence of antibiotic selection antigen expression was rapidly lost for all the clones except the selected clone *M. smegmatis*[pWB106] (Figure 5B). The percentage of cells retaining antibiotic resistance in the absence of selection mirrored the antigen expression data (Figure 5C).

It is known that when bacteria suffer stress both the colony and cellular morphology can change[42]. We observed that the slow-growing recombinants appeared as unusual, smooth, compact and slightly conical colonies, whereas the fast-growers adopted a flat, wrinkled form. To determine if antigen expression affected the cellular morphology, the recombinant *M. smegmatis* were viewed under a fluorescent microscope, where correctly folded GFP could be visualised (Figure 5).

**Figure 6:**
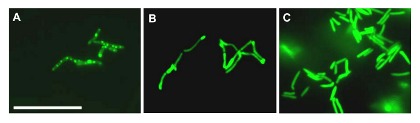
Expression of GFP by A. *M. smegmatis*[pWB105], B. *M. smegmatis*[pWB106. C. *M. smegmatis*[pWB107]. Bar  = 20 µm.

The Gag-GFP fusion protein expressed by *M. smegmatis*[pWB105] was found in granulated aggregates possibly representing inclusion bodies (Figure 6A). The fusion protein expressed from *M. smegmatis*[pWB106] was also highly aggregated, however the fluorescent patches were only located at the ends of the bacilli with possibly more soluble protein expressed, as the entire bacillus had a slightly higher level of fluorescence in comparison to *M. smegmatis*[pWB105] bacilli (Figure 6B). It should be noted that the fluorescence reflects only where properly folded GFP is found, so there may be aggregates of miss-folded antigen that are not identified. The GFP expressed from *M. smegmatis*[pWB107] was uniformly located through-out the cell as would be expected for a properly folded, soluble protein (Figure 6C).

### GFP- and Gag-specific T cell responses after rBCG immunization

**Figure 7:**
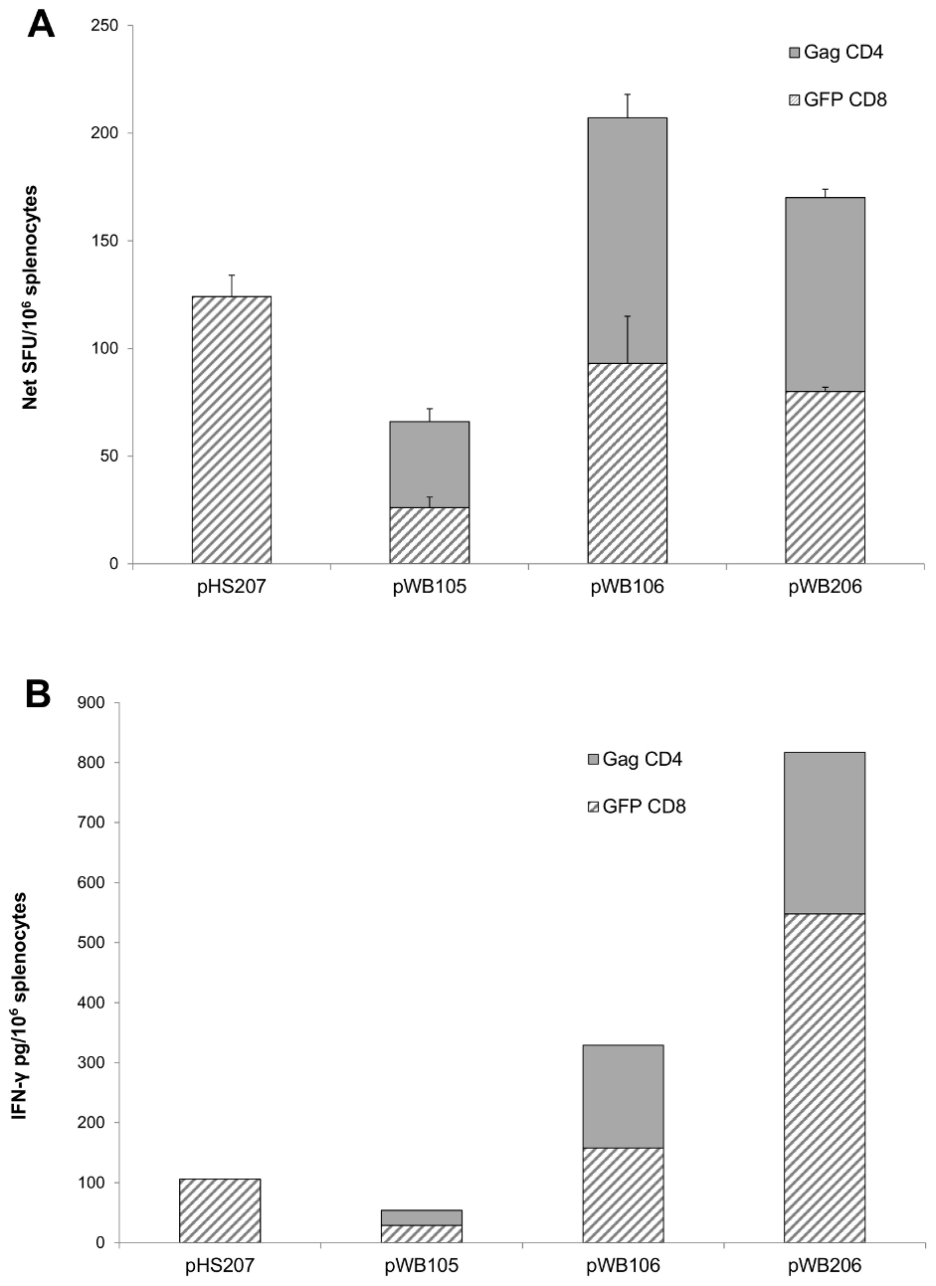
GFP and Gag-Specific immune responses induced by rBCG vaccines. Mice were vaccinated with BCG[pHS207], [pWB105], [pWB106] and [pWB206] (intranasal, 10^7^ CFU). Splenocytes pooled from a group of 5 mice were used on day 56 in A. an IFN-γ ELISPOT assay with the GFPCD8 peptide, GagCD8 peptide or GagCD4 peptide. Bars represent the average number of SFU/10^6^ splenocytes ± the standard deviation of triplicate reactions after subtraction of average background responses of not more than 20±10 SFU per 10^6^ splenocytes. No response to the GagCD8 peptide was detected. B. Splenocytes were cultured (48 h) with the GFPCD8 peptide, GagCD8 peptide or GagCD4 peptide and cytokine levels in the culture supernatant were quantified using a Th1/Th2 cytokine bead array assay and flow cytometry. Only IFN-γ was detected in the culture fluid and for splencoytes stimulated with the GFPCD8 and GagCD4 peptides. Levels are expressed as pg/10^6^ splenocytes and are from a representative experiment.

**Figure 8:**
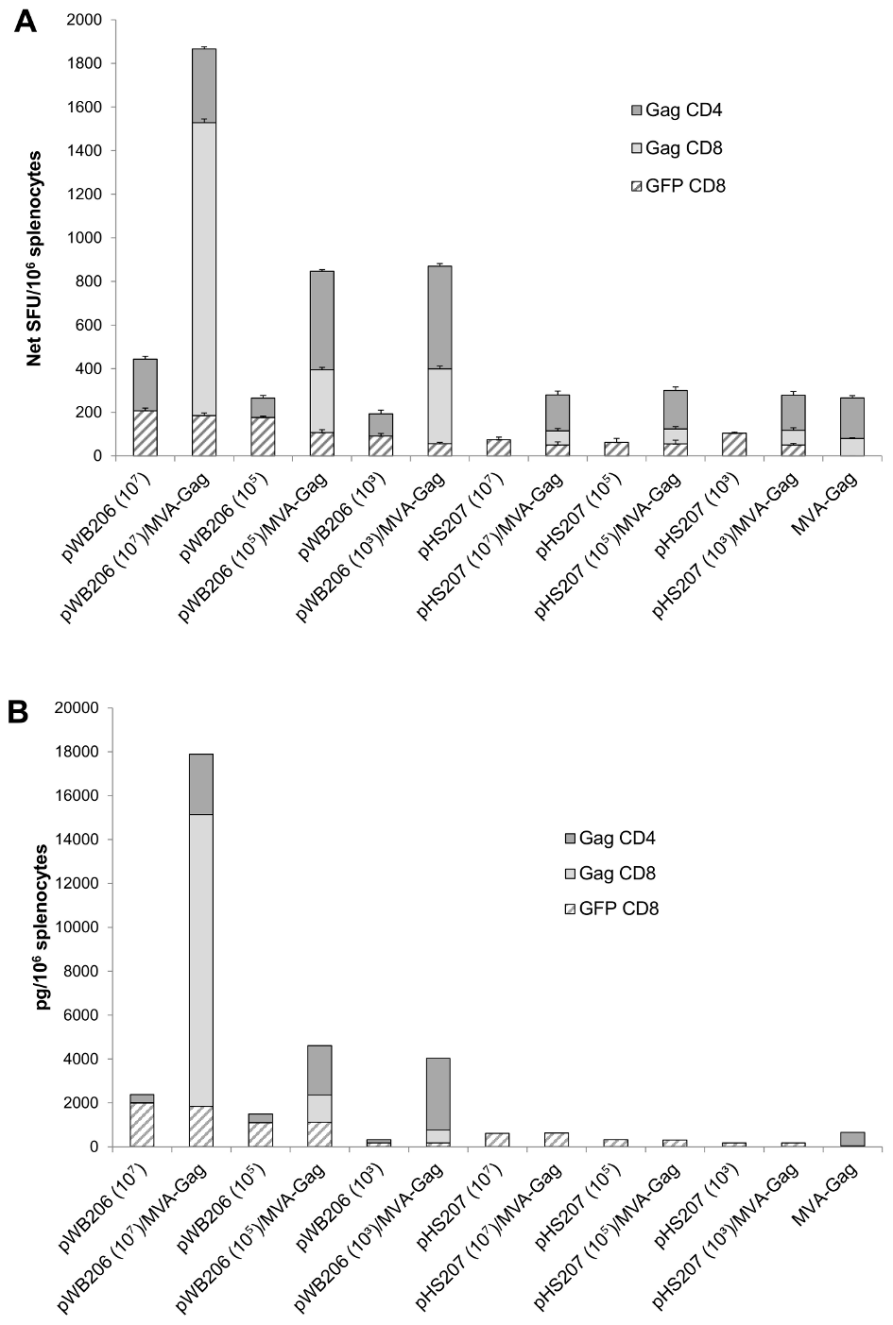
rBCG prime and MVA-Gag boost immune responses. Mice were primed with BCG[pWB206] or BCG[pHS207] (intraperitoneal vaccination; doses of 10^7^ CFU, 10^5^ CFU, 10^3^ CFU) or left unprimed, then boosted on day 56 with MVA-Gag (intramuscular vaccination, 10^7^ pfu). Splenocytes pooled from a group of 5 mice were used on day 68 in **A.** an IFN-γ ELISPOT assay with the GFPCD8 peptide, GagCD8 peptide or GagCD4 peptide. Bars represent the average number of SFU/10^6^ splenocytes ± the standard deviation of triplicate reactions after subtraction of average background responses of not more than 20±10 SFU per 10^6^ splenocytes. **B.** Splenocytes were cultured (48 h) with the GFPCD8 peptide, GagCD8 peptide or GagCD4 peptide and cytokine levels in the culture supernatant were quantified using a Th1/Th2 cytokine bead array assay and flow cytometry. Only IFN-γ was detected in the culture fluid. Levels are expressed as pg/10^6^ splenocytes and are from a representative experiment.

IFN-γ ELISPOT responses 8 weeks after vaccination with BCG[pHS207], [pWB105], [pWB106] and [pWB206] (10^7^ CFU/mouse) indicated that all of these vaccines induced GFP-specific CD8 T cells and for those vaccines expressing modified Gag antigen, Gag-specific CD4 T cells but no Gag-specific CD8 T cells were detected. The range in the cumulative responses to the GFP and GagCD4 peptides was 66–207 SFU/10^6^ splenocytes for the four vaccines (Figure 7A). The Th1/Th2 bias of the immune response to these vaccines was assessed using a cytokine bead array assay to quantify Th1 and Th2 cytokines in the culture medium collected from splenocytes stimulated with the GFP CD8 and the Gag CD4 peptide pool. IFN-γ but no TNF-α, IL-4 or IL-5 was detected (Figure 7B). A high cumulative level of 329 pg IFN-γ/10^6^ splenocytes for vaccine BCG[pWB106] and 817 pg IFN-γ/10^6^ splenocytes for vaccine BCG[pWB206] was produced by stimulation with the GFP CD8 and Gag CD4 peptides. Splenocytes from BCG[ pHS207] and BCG[pWB105] vaccinated mice produced a much lower level of IFN-γ (106 and 71 pg/10^6^ splenocytes respectively) when stimulated with these peptides. The potency of the vaccines BCG[pWB106] and BCG[pWB206] was ranked by relating the frequency of responding cells in the IFN-γ ELISPOT assay which appeared to be similar for these vaccines (Figure 8A) to the level of IFN-γ released from splenoyctes during peptide stimulation (Figure 7B). BCG[pWB206] appeared to be more potent than BCG[pWB106] as GFP CD8 specific T cells together with Gag-specific CD4 T cells produced 4.8 pg IFN-γ per vaccine induced cell whereas BCG[ pWB106] produced 1.5 pg IFN-γ per vaccine induced cell.

### BCG[pWB206] primes the immune system to a boost with MVA-Gag

Priming with one vaccine vector and boosting with another expressing the same HIV antigens provides enhanced cellular immune responses in animal models, thus the ability of BCG[pWB206] to prime the immune response to a boost with MVA-Gag was investigated. Mice were primed with escalating doses of BCG[pWB206], then boosted 8 weeks later with MVA-Gag, and immune responses were assayed 12 days later. Mice primed with BCG[pHS207], which does not express Gag, then boosted with MVA-Gag as well as mice that received only MVA-Gag served as controls in the experiment. The frequency of Gag-specific CD8 and CD4 T cells induced by MVA-Gag was not modulated by a prime with the control vaccine BCG[pHS207] (Figure 8A) nor did MVA-Gag boost the frequency of GFP-specific CD8 T cells induced by the control vaccine pHS207 (Figure 8A). IFN-γ ELISPOT analysis indicated the cumulative frequency of GFP-specific CD8 and Gag-specific CD4 T cells increased in response to increasing doses of BCG[pWB206] (Figure 8A). Although no Gag-specific CD8 T cells were detected at any dose of BCG[pWB206], mice primed with 10^7^ CFU BCG[pWB206] and then boosted with MVA-Gag developed Gag-specific CD8 T cells with a frequency of 1343±17 SFU/10^6^ splenocytes, 16 fold above that induced by MVA-Gag alone. Lower Gag-specific CD8 T cell frequencies of approximately 4 fold above that of MVA alone were observed when mice were primed with doses of 10^5^ and 10^3^ CFU of BCG[pWB206] and then boosted with MVA-Gag (Figure 8A). The frequency of Gag-specific CD4 T cells induced by BCG[pWB206] was boosted 2.5 fold by MVA-Gag for doses of 10^5^ and 10^3^ CFU.

The Th1 bias of the immune response to vaccination with pWB206 was maintained and enhanced by a MVA-Gag boost with the level of IFN-γ produced increasing with higher doses of BCG[pWB206] used as a prime (Figure 8B). Gag-specific CD8 T cells produced 74% of the cumulative IFN-γ of 17900 pg/10^6^ splenocytes in response to a prime with 10^7^ CFU pWB206 and MVA-Gag boost. This is 7.5 fold above the cumulative IFN-γ produced by a vaccination with 10^7^ CFU BCG[pWB206] only (Figure 8A and 8B).

### CTL responses induced by a pWB206 prime and MVA-Gag boost

**Figure 9:**
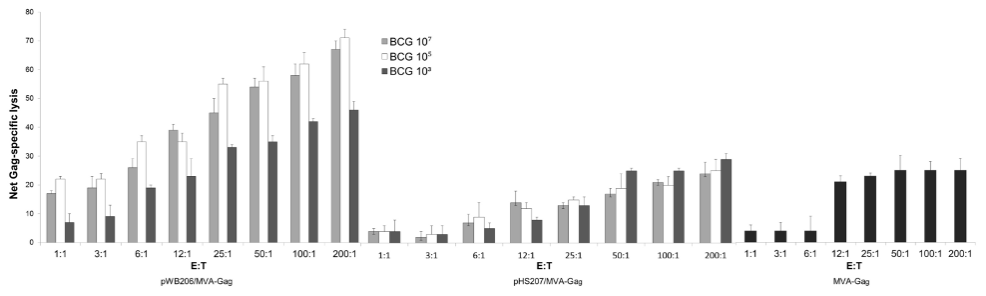
CTL responses measured in a ^51^Cr release assay. Splenocytes pooled from a group of 5 mice on day 68 after a prime with BCG[pWB206] or BCG[pHS207] (10^7^ CFU, 10^5^ CFU, 10^3^ CFU) or no prime and boost with MVA-Gag (10^7^ pfu) on day 56, were stimulated with the GagCD8 peptide for 6 days. Generated effector cells were used in a ^51^Cr release assay using p815 antigen presenting cells in the presence and absence of peptide. Data values indicate the mean net percentage Gag-peptide specific lysis ± the standard deviation (n = 3), calculated after the background lysis (<10%) in the absence of peptide has been subtracted. Net Gag-specific lysis was considered positive if >10% and are shown.

Gag-specific CD8 T cells induced by priming with BCG[pWB206] and a boost with MVA-Gag displayed CTL activity as measured in a ^51^Cr release assay (Figure 9). GagCD8 peptide-specific kill appeared to be positively related to BCG[pWB206] dose and was greater than that for MVA-Gag only. The mean GagCD8 peptide-specific lysis at an E:T ratio of 50∶1, was 54%±3% after a prime with BCG[pWB206] and boost with MVA-Gag, and 17±2% in response to a MVA-Gag vaccination alone (Figure 9). A BCG[pHS207] prime and MVA-Gag boost had no effect on GagCD8 peptide-specific kill by MVA-Gag alone (Figure 9).

### The Gag-Specific immune response induced by BCG[pWB206] protects mice against a VVGag C challenge

**Figure 10:**
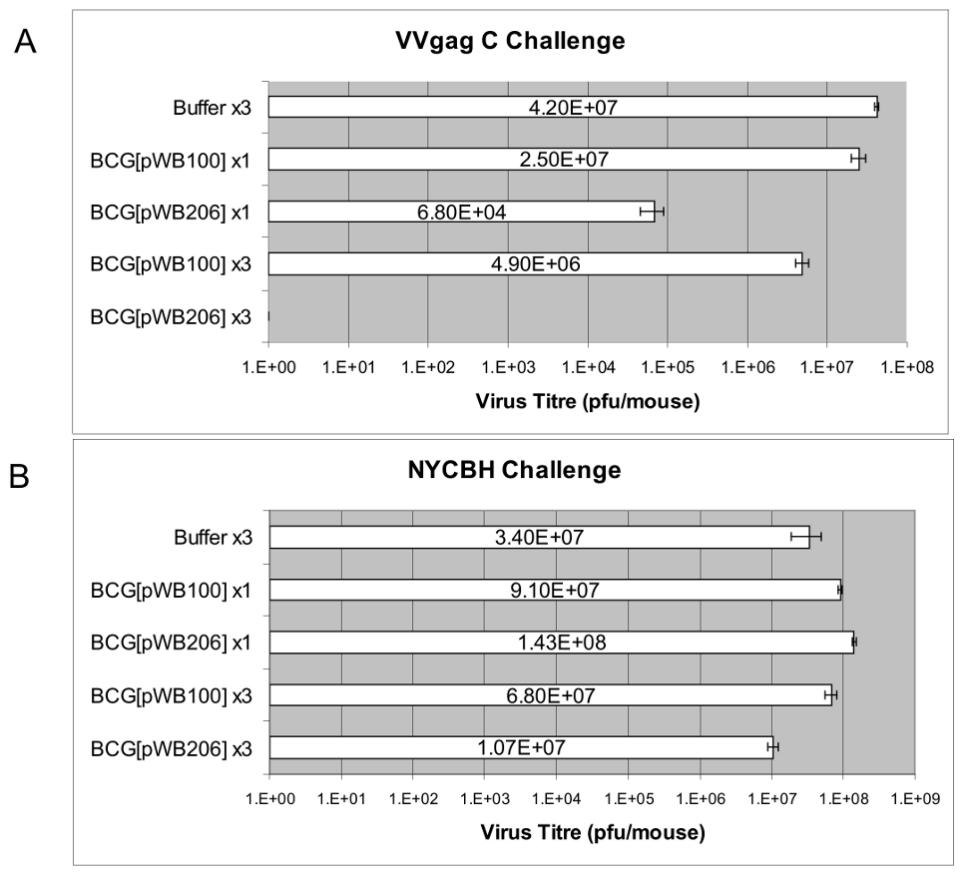
Protection against Vaccinia Virus Gag challenge. BALB/c mice were inoculated intraperitoneally either once or three times with BCG[pWB206], BCG[pWB100], or resuspension buffer. Triple inoculations were given at 4 week intervals. The mice were intraperitoneally challenged with **A.** VV-Gag or **B.** the wild type vaccinia virus strain NYCHB two weeks after the final inoculation. Five days after challenge ovaries were harvested and pooled for each group. The ovaries were processed and virus titres determined. Titrations were performed in triplicate and averaged.

BALB/c mice were inoculated intraperitoneally either once or three times with BCG[pWB206], BCG[pWB100] or resuspension buffer. Triple inoculations were given at 4 week intervals. Mice were intraperitoneally challenged with VV-GagC two weeks after the final inoculation. Five days later ovaries were harvested, pooled and the VV-GagC titres determined. The Gag-specific CD8 T cells elicited by the BCG[pWB206] vaccine clearly provided protection from a challenge with VV-GagC (Figure 10). A single inoculation of 2×10^6^ CFU reduced the VV-GagC titre by approximately 2 logs, while three inoculations of 2×10^6^ CFU resulted in complete (greater than 5 log) protection against VV-GagC. This suggests that homologous boosting is effective and does not impact negatively on the immune response.

Challenge of all groups of mice with the control virus NYCBH resulted in the recovery of similar titres of VV from the ovaries. In addition there was no inhibition of virus growth detected in mice inoculated with buffer or the control rBCG prior to challenge with VVGag C.

## Discussion

This study has shown that growth rate of mycobacteria is reduced as recombinant HIV-1 antigen expression increases and that this reduced fitness is the driving force behind recombinant instability. We argue that the expression of miss-folded antigen in *M. smegmatis* causes induction of stress response. This serves to explain the often poor expression of recombinant protein in mycobacteria. Usually, it is assumed that there is some form of bottle-neck in protein synthesis; however, the level of protein is determined by the balance between production and destruction, so probably the low level of Tat-Gag protein is due to accelerated degradation, caused by the fact that it is miss-folded. This model explains why, when a protein is expressed in a closely related host, extremely high levels of expression can usually be achieved and the expression vector is stable, while the converse is often true when a protein from a distantly related organism is expressed. Thus, to increase antigen expression it seems necessary to express the antigen in a manner that is acceptable to the bacterial quality control mechanism. We have utilised a directed evolution approach to create recombinant mycobacteria that show both improved antigen expression and fitness. The recombinants were shown to be more stable and to generate immune responses that varied, in a manner dependent upon antigen expression levels.

Immune responses of mice to BCG[pWB105], BCG[pWB106] and BCG[pWB206] with a range of Gag p24 expression levels indicate that the magnitude of immune response to Gag may be related to the level of recombinant antigen expressed (Figure 8A). Both GFP CD8 and Gag CD4 T cells specific for the insert were generated. The absence of Gag CD8 T cell detection was probably due to the immunodominance of the GFP CD8 epitope. Although BCG *per se* induces CD4 T cell responses in mice, our observation of induction of insert specific GFP CD8 and Gag CD4 T cells suggests that both MHC class I and II presentation of the antigen occurs. The generation of CD8 T cells is possibly a consequence of efficient cross priming [43], [44]. Generally, responses of mice to a recombinant antigen carried by BCG are predominantly CD4 specific, while insert specific CD8 cells are generated when the antigen is carried by *M. smegmatis* [45]–[47]. The very low overall cellular responses to BCG[pWB105] may be associated with low Gag p24 expression levels. For BCG[pWB206] where Gag p24 expression levels were greater than that of BCG[pWB106], similar frequencies of GFP- and Gag-specific IFN-γ producing T cells were detected for both vaccines. The major difference in the immune responses to these two vaccines appears to lie in the capacity of the induced T cells to produce IFN-γ. The GFP CD8 and Gag CD4 T cells induced by pWB206 had a higher IFN-γ producing capacity than that of BCG[pWB105]. The nature of the Gag p24-GFP protein being expressed by the recombinants may account for this, with the ratio of soluble to insoluble protein playing a role. It is important to note that although we could not detect effector Gag CD8 T cells, memory cells must have been induced by BCG[pWB206] vaccination, as inhibition of infection by vaccinia virus expressing Gag was observed.

BCG[pWB206] possessed the potential to prime the immune system to a boost with MVA-Gag. This is in agreement with other studies in which HIV antigen-expressing recombinant *M. bovis* BCG and *M. smegmatis* were used in heterologous regimens with recombinant viral or protein vaccines as a boost [15], [16], [45]–[52]. Both Gag-specific CD8 and CD4 T cells with a high capacity to produce IFN-γ at levels above those of the individual vaccines were detected after the boost. In addition, the magnitude of these Gag-specific CD8 and CD4 T cells was dose dependent. These responses were specific and not due to non-specific immune activation occurring through BCG[pWB206] enhancing secondary immune responses, as no non-specific Gag priming occurred with BCG[pHS207]. Expansion of Gag CD8 T cells in response to a boost with MVA-Gag is a further indication that memory CD8 T cells are induced by a BCG[pWB206] prime. Again, and is in agreement with other studies, where memory T cells induced by recombinant *M. bovis* and *M. smegmatis* were shown to expand following an MVA boost [15], [45], [53]. Even low doses of BCG[pWB206] induces a sufficient frequency of Gag-specific CD8 memory cells to be detectable upon MVA boosting. This generation of Gag-specific memory CD8 T cells by the prime may occur as a consequence of help from the prime-induced Gag-specific CD4 T cells that we detect [54], [55]. In addition, the slow doubling times of BCG result in reduced levels of antigen generated followed by poor antigen presentation and subsequently delayed and weak priming of CD8 T cells which differentiate primarily into the central memory CD8 T cell subset. These CD8 memory cells are able to proliferate rapidly and produce cytokines after boosting [56], [57]. The Gag-specific CD8 T cells induced by the boost possessed cytolitic activity. Although the nature of the immune responses required for a vaccine aimed at the prevention of HIV infection are not clear, Gag-specific responses by HIV infection in humans is associated with the level of infection control [58], [59]. Complete protection against a surrogate virus challenge was provided by three doses of BCG[pWB206] alone, however, we were not able to investigate this after a single BCG[pWB206] prime-MVA-Gag boost due to vector immune response interference.

In this study we have shown that the stability and levels of recombinant antigen expression of BCG expressing HIV-1 Gag can be improved through modification of the antigen. Recombinant BCG expressing the modified HIV-1 Gag, were able to induce HIV-specific T cells that protected against a surrogate vaccinia virus challenge and were strongly boosted by MVA-Gag.
